# Sex-informed estrogen receptor modulation in schizophrenia: a male-focused ERβ/GPER1 framework for cognitive and negative symptoms

**DOI:** 10.3389/fpsyt.2025.1714569

**Published:** 2026-02-09

**Authors:** John Carlson

**Affiliations:** Independent Researcher, Bozeman, MT, United States

**Keywords:** adjunctive therapy, ERβ, estrogen receptors, GPER1, neuroprotection, schizophrenia, selective estrogen receptor modulators

## Abstract

Treatment-resistant schizophrenia (TRS) remains a major unmet clinical need, particularly in males who exhibit more severe negative and cognitive symptoms and limited responsiveness to dopamine-based therapies. Estrogenic signaling—especially through estrogen receptor beta (ERβ) and the G-protein–coupled estrogen receptor 1 (GPER1)—has emerged as a promising neuromodulatory target for these domains. This review synthesizes evidence supporting selective estrogen receptor modulators (SERMs), with an emphasis on raloxifene, as adjunctive agents capable of engaging central estrogenic pathways without feminizing systemic effects. Preclinical, stem cell–derived, and clinical data demonstrate that ERβ- and GPER1-mediated signaling enhances synaptic plasticity, mitochondrial stability, anti-inflammatory glial states, and dopaminergic–glutamatergic balance—core processes implicated in TRS pathophysiology. Complementary findings involving aromatase activity, neurosteroidogenesis, and genetic variation in ESR2 and CYP19A1 highlight opportunities for biomarker-guided stratification. The review also addresses ethical and gender-inclusive considerations, framing estrogenic modulation as sex-informed but not sex-restricted, given the universal expression of ERβ and GPER1 across sexes. Finally, emerging innovations—including GPER1-biased ligands, tissue-selective estrogen complexes, and nanoparticle delivery systems—are discussed as strategies to optimize central nervous system targeting while minimizing peripheral risk. Together, these insights position receptor-selective estrogenic modulation as a mechanistically grounded and clinically relevant approach for improving cognitive and negative symptom outcomes in TRS.

## Introduction

Treatment-resistant schizophrenia (TRS), defined by insufficient response to at least two adequate antipsychotic trials, affects up to one-third of individuals with the disorder (1, 2). Males often show earlier onset, higher rates of persistent negative symptoms, and poorer functional recovery, although these patterns are heterogeneous and not universal (3). These clinical features point toward broader pathophysiological contributions—particularly oxidative stress, glutamatergic imbalance, and neuroimmune dysregulation—that are insufficiently addressed by D2 dopamine receptor (DRD2) blockade (1).

We propose that a major component of TRS may arise from underrecognized dysfunction in estrogenic signaling. Estradiol, acting through estrogen receptor beta (ERβ) and the G-protein–coupled estrogen receptor (GPER1), regulates synaptic plasticity, mitochondrial function, and microglial reactivity—domains consistently disrupted in schizophrenia (4, 5). Both receptors are expressed in male corticolimbic circuits, and preclinical studies show that their downstream effects can be protective across sexes in many model systems, although important sex differences in responsivity have also been documented and must be acknowledged (6, 7).

Clinically, raloxifene has demonstrated improvements in cognition and negative symptoms in schizophrenia, including in refractory populations; however, findings are mixed. Notably, the most recent randomized trial (8) reported detrimental working-memory effects in men alongside beneficial effects in women, underscoring the need for sex-informed interpretation (9).

This article advances the hypothesis that TRS can be reconceptualized as a receptor-level mismatch: dopamine blockade alone is insufficient, and adjunctive modulation of ERβ and GPER1 may address neural domains resistant to antipsychotics. SERMs, through selective activation of intracellular cascades relevant to synaptic and immune regulation, therefore represent a mechanistically grounded yet exploratory strategy for targeting refractory cognitive and negative symptoms within a sex-informed but inclusive framework.

Standard antipsychotics, which primarily modulate dopaminergic signaling, exert limited influence on glutamatergic dysfunction, oxidative stress, and immune imbalance. These broader abnormalities have catalyzed renewed interest in alternative neuromodulatory systems—most prominently the estrogenic network, recognized for its regulation of plasticity, mitochondrial homeostasis, and synaptic stability (10).

Our aim is not to frame schizophrenia as a hormonally driven disorder, but to position estrogenic modulation as a precision adjunct embedded within a broader multi-pathway neurobiological model. To support this framework, we integrate evidence from receptor pharmacology, stem cell–derived neural models, preclinical studies, and clinical trials, and outline future-direction strategies—including GPER1-biased ligands, brain-selective SERMs, and nanoparticle delivery systems—that may inform the next generation of estrogenic approaches in psychiatry.

## Hypothesis statement

We hypothesize that treatment-resistant schizophrenia arises in part from insufficient engagement of ERβ- and GPER1-dependent estrogenic signaling within corticolimbic circuits.

Specifically:

1. ERβ and GPER1 regulate synaptic, mitochondrial, neuroimmune, and glutamatergic pathways disrupted in TRS.
2. Males may exhibit heightened vulnerability in these pathways due to developmental, endocrine, and stress-related factors, though the mechanism itself is not sex-exclusive.
3. Adjunctive activation of ERβ and GPER1—particularly via SERMs—may restore circuit-level function not addressed by dopamine blockade alone.

## Aromatase expression and neurosteroidogenesis in schizophrenia

Aromatase (CYP19A1)—the enzyme responsible for converting androgens into estrogens—is expressed across corticolimbic regions including the hippocampus, prefrontal cortex, and amygdala, all of which show structural and functional alterations in schizophrenia (11). Although direct evidence in schizophrenia remains limited, region- and sex-dependent differences in CYP19A1 activity may influence local estradiol availability. Such variation affects both genomic estrogen receptors (ERα and ERβ) and rapid non-genomic signaling cascades that interact with dopaminergic and glutamatergic pathways implicated in the disorder (5).

Estrogen also helps regulate neuroimmune and redox processes. Inflammatory conditions can increase cerebellar estradiol synthesis during early development (12), suggesting that CYP19A1 may function as a context-dependent neuroprotective buffer. Complementary neurosteroid literature indicates that locally synthesized estrogens modulate glutamatergic tone and synaptic plasticity—mechanisms relevant to psychiatric risk and resilience, even if not specific to schizophrenia (13).

Findings from neurology further support the broader plausibility of this framework. In Parkinson’s disease, interactions among sex hormones, sex chromosomes, and neuroinflammatory signaling have been implicated in shaping disease trajectories, illustrating the widespread relevance of estrogenic modulation to neural stability (14). Astrocyte-derived estradiol similarly regulates reactive astrogliosis and enhances neuroprotection after ischemic injury (15). While these mechanisms originate outside schizophrenia, they converge on the principle that local estradiol synthesis can mitigate forms of neural stress across conditions.

Taken together, these observations suggest that genetic variation in CYP19A1 or inflammation-driven modulation of CYP19A1 activity may contribute to individual differences in schizophrenia vulnerability and illness progression. Altered neurosteroidogenesis thus represents one potential contributor to treatment resistance, and aromatase-related biomarkers may ultimately support stratification for receptor-targeted adjunctive interventions.

## Adjunctive SERM + estradiol therapy: cognitive and negative symptom targets

Conventional antipsychotics primarily reduce positive symptoms through dopaminergic blockade, yet provide limited benefit for cognitive deficits and negative symptoms. This therapeutic gap has motivated interest in estrogenic neuromodulation using SERMs, particularly raloxifene. Acting through ERβ and GPER1, SERMs influence neuroplasticity, affect regulation, and synaptic efficiency—domains impaired in schizophrenia (16, 17).

Clinical findings support these mechanistic targets. In a 24-week randomized controlled trial, adjunctive raloxifene improved verbal memory and executive function in postmenopausal women with schizophrenia (18). Genotype-sensitive neuroimaging work further demonstrates increased prefrontal activity during emotional inhibition following SERM treatment (19), and estrogen receptor genotype appears to shape these responses (20). A meta-analysis including both sexes found that raloxifene augmentation can improve negative symptoms and enhance antipsychotic response in schizophrenia-spectrum disorders (2). Functional MRI studies also report increased hippocampal and inferior frontal activation during emotional processing under SERM therapy, linking receptor engagement to recruitment of cognitive circuits (21).

Mechanistic evidence aligns with these clinical observations. Estrogen and SERMs enhance synaptic plasticity through brain-derived neurotrophic factor (BDNF) signaling (22), promote mitochondrial stability and bioenergetic efficiency (23), and activate PI3K/Akt–CREB pathways central to learning and memory (24, 25). Neuroimmune processes may also contribute: estrogenic signaling promotes pro-resolving microglial phenotypes (16), and inflammation is strongly associated with negative symptom severity and motivational deficits (26). Additional work implicates redox buffering and metabolic regulation as contributors to sustained cognitive effects (23, 27).

However, findings remain mixed and context-dependent. The most recent randomized controlled trial (8) reported detrimental working-memory effects in men and beneficial effects in women, highlighting the importance of sex, age, hormonal milieu, and receptor-genetic variation in shaping treatment response. Large-scale estradiol trials likewise show improvements in selected treatment-resistant cases but not uniformly across populations (28).

Taken together, these converging mechanistic and clinical findings suggest that adjunctive SERM–estradiol strategies may offer targeted benefits for cognitive and negative symptoms—domains insufficiently addressed by dopamine-focused treatments—by integrating trophic, metabolic, and immunoregulatory effects within a precision psychiatry framework.

## Estrogen-linked mechanisms of neural protection in schizophrenia

Estrogens act as pleiotropic neuromodulators with anti-inflammatory, antioxidant, and neurotrophic effects—domains disrupted in schizophrenia (24, 29). Across animal, cellular, and human studies, estradiol enhances synaptic plasticity, stabilizes mitochondrial energetics, and activates redox-sensitive transcriptional pathways relevant to neuropsychiatric resilience. A central mechanism involves the induction of BDNF, which is frequently reduced in schizophrenia and is sensitive to estrogenic regulation (22, 30).

Although the present framework emphasizes ERβ and GPER1 due to their relevance for male TRS, ERα also contributes to estrogenic neuroprotection. ERα-mediated genomic signaling influences plasticity, oxidative regulation, and immune modulation in several preclinical models, and its partial engagement by SERMs such as tamoxifen provides complementary mechanistic pathways that may interact with ERβ- and GPER1-driven effects. Acknowledging ERα activity strengthens the completeness of the mechanistic landscape while retaining the receptor-selective focus of this work.

Estrogen also modulates neuroimmune processes. Through ERα, ERβ, and GPER1, estradiol promotes pro-resolving microglial phenotypes and suppresses pro-inflammatory cytokine activity (16), countering the heightened inflammatory tone—that is, the overall cytokine burden and pro-inflammatory signaling state—observed in subsets of patients. At the mitochondrial level, estradiol reduces oxidative stress and preserves synaptic and bioenergetic function (23), with downstream effects mediated in part by PI3K/Akt–CREB and MAPK/ERK signaling pathways that support synaptogenesis and cytoprotection (25, 31).

**Figure 1:**
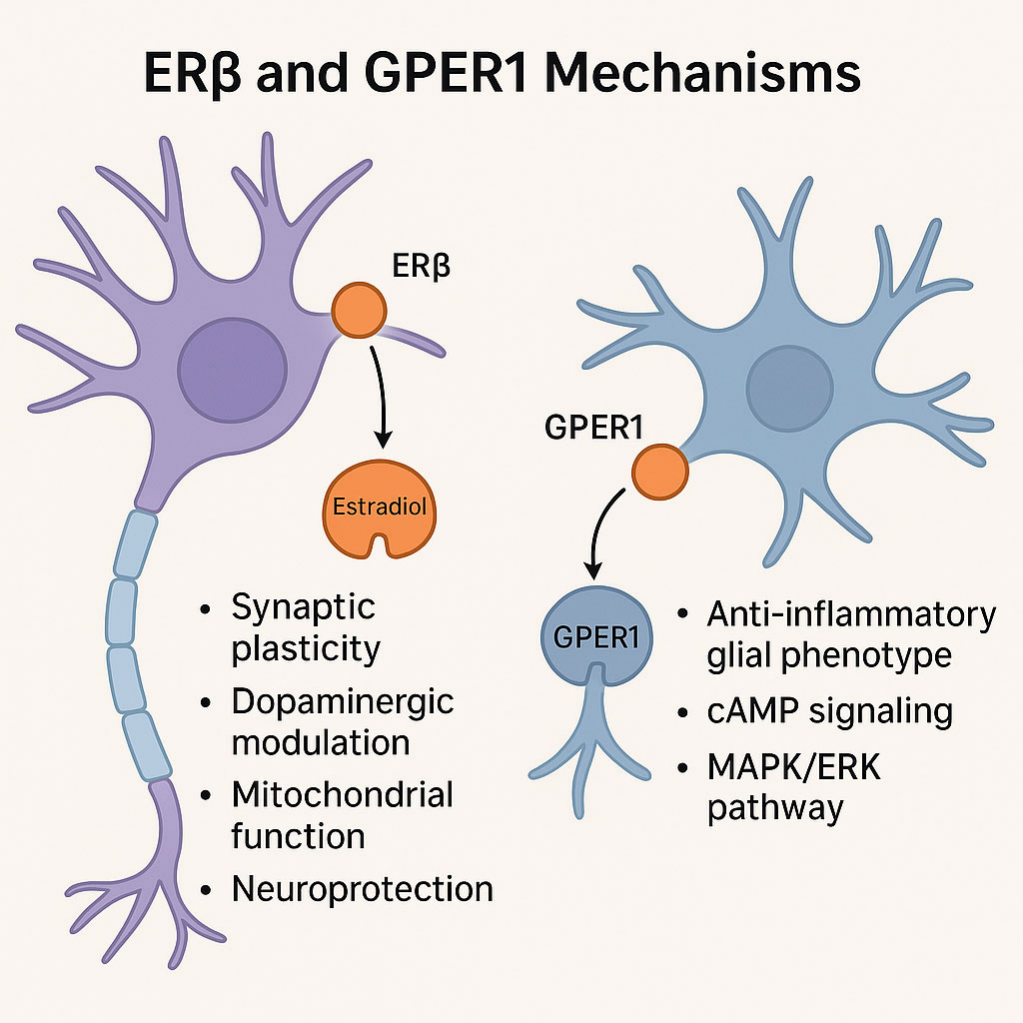
Integrated ERβ/GPER1 mechanistic model in TRS.

Findings from induced pluripotent stem cell (iPSC)-derived models and cerebral organoids further demonstrate that estradiol enhances neuron–glia communication and cortical network organization, mechanisms increasingly implicated in schizophrenia pathophysiology (6). Taken together, these actions converge on synaptic remodeling (22), immune regulation (16), and redox homeostasis (23)—core domains affected in schizophrenia—supporting estrogenic modulation as a biologically grounded adjunctive approach for neuroprotection and cognitive enhancement. **Figure 1** illustrates complementary ERβ- and GPER1-mediated signaling pathways relevant to treatment-resistant schizophrenia.

## GPER1 and ERβ: dual pathways in affective and executive circuitry

GPER1 and ERβ modulate affective regulation and executive functioning—two domains prominently impaired in schizophrenia. Although these receptors initiate distinct intracellular cascades, both converge on processes regulating synaptic stability, immune activity, mitochondrial function, and neurotransmission within corticolimbic circuits.

GPER1, a membrane-associated receptor expressed in the medial prefrontal cortex, hippocampus, and amygdala, rapidly activates ERK1/2 and PI3K/Akt pathways to support dendritic complexity, synaptic maintenance, and mitochondrial stability (32, 32). In parallel, GPER1 signaling promotes pro-resolving microglial phenotypes (16) and mitigates stress-related neurotoxicity, with additional evidence showing protection against haloperidol-induced cellular injury and improvement of neural function in preclinical models (34).

ERβ, often co-expressed with GPER1, regulates transcriptional programs involved in synaptogenesis, glutamatergic signaling, oxidative resilience, and long-term neuronal maintenance. ERβ contributes to BDNF induction (22), supports inhibitory balance through GABAergic and serotonergic pathways (35), and is sensitive to developmental endocrine and inflammatory influences that shape hypothalamic–pituitary–adrenal (HPA) axis responsivity and affective processing (35). Sex differences in ERβ responsivity have been documented across clinical and preclinical models, emphasizing the need for sex-informed but inclusive interpretation of these mechanisms (36, 37).

Together, GPER1 and ERβ act synergistically to stabilize excitatory–inhibitory balance, astroglial signaling, and neuroinflammatory responses under oxidative stress. This integrated receptor framework strengthens the rationale for dual-target SERMs as potential adjuncts for executive and affective dysfunction in schizophrenia (36, 37).

Core symptoms of schizophrenia arise in part from dysregulated dopaminergic and glutamatergic signaling within prefrontal–limbic pathways. Estrogen receptors—particularly ERβ and GPER1—modulate these systems through mechanisms that may counter both subcortical hyperdopaminergia and reduced mesocortical dopaminergic drive. Estradiol influences tonic and phasic dopamine release, enhances mesocortical dopamine signaling, and interacts with motivational and reward pathways (38). These processes are relevant to negative and cognitive symptoms that remain poorly responsive to D2 antagonism.

At the glutamatergic level, ERβ- and GPER1-dependent signaling modulates N-methyl-D-aspartate (NMDA) and α-amino-3-hydroxy-5-methyl-4-isoxazolepropionic acid (AMPA) receptor activity, facilitating synaptic strength (22), long-term potentiation (39), and plasticity within hippocampal and limbic circuits (22, 33). Estradiol also protects against glutamate-induced excitotoxicity through PI3K/Akt and MAPK/ERK pathways (25, 39) while stabilizing mitochondrial dynamics and reducing oxidative stress (23).

Collectively, these receptor-mediated actions suggest that estrogenic modulation may help restore excitatory–inhibitory balance and influence core circuit dysfunction relevant to treatment-resistant schizophrenia—although current evidence remains preliminary and shaped by sex, age, and receptor-genetic variation (36).

## Immunomodulatory effects of estrogen in psychosis-related inflammation

Neuroimmune dysregulation contributes to schizophrenia in a subset of individuals, with abnormalities in cytokine profiles, glial signaling, and oxidative stress linked to motivational deficits, negative symptoms, and cognitive impairment (26, 40). Importantly, current evidence indicates that these immune abnormalities occur in only a subset of patients—rather than representing a universal feature of schizophrenia or TRS. Estrogens—acting through ERβ and GPER1—modulate these pathways by reducing pro-inflammatory cytokines, enhancing microglial clearance functions, and promoting pro-resolving immune phenotypes (16, 20). Rather than adopting discrete “M1/M2” states, microglia exist on a multidimensional activation spectrum; estrogenic signaling is associated with shifts toward less pro-inflammatory, more homeostatic phenotypes relevant to neural circuit maintenance (16).

Beyond cytokine regulation, estradiol enhances redox balance and mitochondrial stability, limiting oxidative cascades that potentiate glial overactivation and excitotoxic vulnerability (23, 30). These effects extend to astrocyte–neuron communication: in iPSC-derived neural models, estradiol improves neuron–glia signaling and supports cortical network organization, suggesting potential mechanistic relevance to the synaptic and inflammatory abnormalities observed in schizophrenia (6).

Inflammatory stimuli can also modulate CYP19A1 expression, enabling localized neurosteroidogenesis during neural stress. Pro-inflammatory signals such as prostaglandin E2 and interleukin-1 beta (IL-1β) increase glial CYP19A1 activity in the cerebellum and hippocampus (12, 41), facilitating local estradiol synthesis as a context-dependent compensatory response. Although not specific to schizophrenia, these mechanisms illustrate how inflammatory states may dynamically alter estrogen availability within the brain.

These pathways have particular relevance for individuals with treatment-resistant schizophrenia, some of whom exhibit elevated oxidative stress or inflammatory markers (27, 42). However, such immune abnormalities are not universal and may be influenced by illness severity, antipsychotic exposure, or metabolic comorbidity (e.g., antipsychotics themselves can elevate inflammatory markers) (40, 42). Accordingly, estrogenic modulation should be viewed as a potential adjunct targeting specific immune-related subgroups rather than a uniform mechanism across all TRS presentations.

Taken together, current evidence suggests that ERβ- and GPER1-associated pathways may help buffer oxidative and inflammatory stressors that interact with cognitive and negative symptom domains. These observations remain preliminary but support continued investigation of estrogenic and aromatase-linked mechanisms as part of a precision framework for identifying inflammation-sensitive schizophrenia subtypes (22, 43). A synthesis of these interactions is provided in **Table 1**.

**Table 1:** Summary of estrogen-linked mechanisms relevant to TRS.

Mechanism/effect	Biological role/pathway	Relevance to schizophrenia	Key references
ERβ activation	Genomic modulation of BDNF, glutamatergic, and GABAergic gene networks	Enhances cognitive flexibility, neurotrophic support, and stress resilience	(22, 35)
GPER1 signaling (PI3K/Akt, MAPK/ERK)	Rapid non-genomic signaling regulating synaptic strength, dendritic structure, and mitochondrial stability	Improves affective regulation, synaptic integrity, and stress adaptation	(32, 33)
CYP19A1 activity	Converts androgens to estrogens in PFC, hippocampus, amygdala; inducible by inflammatory cytokines	Local estrogen synthesis modulates plasticity, neuroimmune tone, and stress-reactivity	(12, 41)
Mitochondrial effects	Enhances ATP production, reduces ROS, stabilizes membrane potential	Counters oxidative stress and bioenergetic deficits linked to cognitive impairment	(23, 30)
Neuroimmune modulation	Reduces interleukin-6 (IL-6) and tumor necrosis factor-alpha (TNF-α); promotes pro-resolving microglial phenotypes (M2)	Limits chronic neuroinflammation associated with TRS and negative symptoms	(16, 40)
Neurogenesis & synaptic plasticity	Stimulates hippocampal neurogenesis, LTP, and synaptic remodeling	Supports memory and cognitive circuits disrupted in schizophrenia	(6, 25)
Dopaminergic modulation	Regulates D2 receptor availability, dopamine synthesis, and mesocortical signaling	May reduce mesolimbic hyperdopaminergia and improve executive function	(35, 38)
Glutamatergic regulation	Enhances NMDA/AMPA signaling, supports excitation–inhibition balance	Relevant to negative symptoms, cognitive deficits, and cortical thinning	

This table integrates mechanistic findings across clinical, preclinical, and in vitro systems. To avoid overgeneralization, each entry identifies whether the supporting evidence derives from:

1. Clinical studies (C)

2. Preclinical animal studies (P)

3. In vitro/organoid/iPSC studies (I)

and specifies the sex examined (M, male; F, female; MIX, mixed/unspecified). Most mechanistic studies are preclinical or mixed-sex and were not conducted in TRS samples; therefore, these findings provide biological rationale, not direct clinical evidence, for SERM use in men with TRS.

Because the majority of mechanistic studies summarized here were performed in non-TRS clinical samples, mixed-sex animal models, or *in vitro* systems, these pathways should be interpreted as providing biological plausibility rather than definitive evidence that SERMs improve cognitive or negative symptoms specifically in men with TRS.

## Clinical trials and meta-analyses of estrogenic interventions in schizophrenia

Over two decades of clinical research have examined adjunctive estrogens and selective SERMs as potential therapeutic strategies in schizophrenia. Early large-scale randomized controlled trials demonstrated that estradiol augmentation can improve positive symptoms in women with treatment-resistant schizophrenia, including meaningful reductions in Positive and Negative Syndrome Scale (PANSS) scores (28). Subsequent meta-analytic work by de Boer and colleagues found evidence that SERMs may exert beneficial effects across sexes, suggesting that some therapeutic actions could arise from receptor-specific mechanisms—particularly ERβ and GPER1 signaling—rather than from endocrine replacement alone (2).

In postmenopausal women, several randomized controlled trials report cognitive and affective improvements with adjunctive raloxifene. For example, a 24-week trial showed enhanced verbal memory and executive function (18), while functional neuroimaging studies demonstrate increased hippocampal and inferior frontal activation during emotional processing under SERM treatment (21). These findings align with preclinical evidence that estrogenic signaling supports synaptic and cognitive networks.

Although clinical evidence in men remains more limited, available data indicate that the requisite receptor biology—ERβ and GPER1 expression in corticolimbic pathways—is present in male patients, supporting the mechanistic plausibility of cross-sex intervention effects (32, 37). Nonetheless, recent trials highlight important sex-specific variability. A large randomized trial in treatment-resistant women found improvements in negative symptoms, though effects on broader psychopathology remained modest (45). Meta-analyses similarly report mixed findings, with some studies observing limited benefits for negative or cognitive symptoms despite strong preclinical rationale (46). The most recent randomized controlled trial of raloxifene in schizophrenia (8) reported beneficial effects in women but detrimental effects on working memory in men, underscoring the need for careful sex-stratified interpretation of clinical outcomes.

These heterogeneous findings reinforce the importance of biomarker-guided approaches. Not all patients appear equally responsive to estrogenic interventions, and subgroup differences may relate to hormonal milieu, receptor distribution, inflammatory status, or genetic variation— including polymorphisms in ESR2 (estrogen receptor beta gene) and COMT (catechol-O-methyltransferase gene), or CYP19A1 (45). Future precision psychiatry trials will likely require such stratification to identify individuals most likely to benefit from SERM- or estradiol-based adjunctive therapies and to clarify the neurobiological subpopulations for whom estrogenic modulation is most clinically appropriate.

**Table 2** Summary of clinical evidence for estrogenic interventions in schizophrenia. This table synthesizes key clinical trials and meta-analyses examining adjunctive estrogenic strategies in schizophrenia, including estradiol augmentation, SERMs such as raloxifene, and mechanistically relevant agents like tamoxifen. For each intervention, population characteristics, targeted symptom domains, and principal findings are summarized. Evidence reflects variability across sex, hormonal milieu, illness stage, and receptor-genetic profiles. The table highlights that while several trials report improvements in cognitive, affective, or negative symptom domains—particularly in women—other studies show modest or null effects, underscoring the need for biomarker-guided stratification in future precision psychiatry approaches.

**Table 2:** Summary of clinical evidence for estrogenic interventions in schizophrenia.

Intervention	Population	Outcome domains	Summary of findings	References
Estradiol augmentation	Premenopausal & postmenopausal women	Positive symptoms, cognition	Several RCTs show improvement; strongest effects in positive symptoms; variable cognitive results	(18, 28)
Raloxifene (adjunct)	Men & women	Cognition, negative symptoms, emotional regulation	Mixed findings; multiple trials show improvements; meta-analyses support moderate effects	(2, 8, 18, 44, 45, 47)
Tamoxifen	Bipolar disorder (cross-diagnostic relevance)	Mania, affect regulation	Reduces manic symptoms; mechanisms relevant to schizophrenia (PKC inhibition, ER signaling)	(48–50)
Estradiol-sensitive biomarkers	Male & female patients	Stratification	Hormonal levels, COMT genotype, and CYP19A1 polymorphisms predict response variability	(46)

## Cross-diagnostic hormonal evidence: from bipolar to schizophrenia

Reproductive and endocrine transitions in bipolar disorder, such as perimenopause and the postpartum period, consistently reveal how fluctuations in estradiol influence mood instability, cognitive control, and affective reactivity (29, 51). These periods of endocrine sensitivity modulate prefrontal–limbic circuits, stress reactivity, and neuroimmune signaling—domains also implicated in schizophrenia—providing a clinically observable example of how hormonal context shapes neuropsychiatric trajectories. While bipolar and schizophrenia are distinct diagnoses, their shared corticolimbic and executive-control circuitry makes bipolar hormonal transitions a useful comparative model for understanding estrogen-linked vulnerability.

Across diagnostic categories, convergent estrogen-regulated mechanisms are repeatedly implicated in symptom severity and treatment responsivity. These include BDNF-dependent plasticity (22), mitochondrial and redox homeostasis (23), and glutamatergic synaptic modulation (24). Such pathways align with key schizophrenia domains—negative symptoms, cognitive impairment, and frontostriatal inefficiency—highlighting a mechanistic bridge rather than an equivalence between disorders.

Translational work further reinforces this mechanistic overlap. In iPSC-derived neurons and cerebral organoids (not patient-derived in most studies), estradiol enhances astrocyte–neuron communication, improves dendritic structure, and restores synaptic organization (6). Parallel findings show that estradiol increases mitochondrial resilience under stress (23) and that ERβ activation modulates oxidative stress, glial tone, and CREB–BDNF signaling (19, 27). These mechanistic signatures map onto processes relevant to both affective and psychotic vulnerability while not implying diagnostic interchangeability.

Evidence from bipolar disorder also illustrates SERM-linked intracellular actions. Tamoxifen, though developed for oncology, reduces manic symptoms in multiple RCTs via both estrogen-receptor modulation and protein kinase C (PKC) inhibition (48, 49). These intracellular pathways—PKC, calcium signaling, and synaptic regulatory cascades—intersect with dopaminergic, glutamatergic, and neuroimmune circuits central to schizophrenia pathophysiology.

Recent cross-diagnostic reviews report that tamoxifen and raloxifene modulate neuroimmune activation, mitochondrial dysfunction, and stress-responsive circuitry across mood and psychotic disorders (52). These shared biological substrates—glial dysregulation, oxidative imbalance, and corticostriatal signaling deficits—suggest that estrogenic modulation targets systems with relevance across diagnoses while still requiring diagnosis-specific evaluation.

In summary, hormonal evidence from bipolar disorder provides a mechanistic reference point rather than a direct clinical analogy. The convergence across estrogen-regulated pathways, intracellular targets, and stress-responsive circuits supports a receptor-centric framework in which SERM-based interventions may hold transdiagnostic mechanistic relevance. This informs schizophrenia-specific hypotheses while respecting diagnostic boundaries and the preliminary nature of cross-diagnostic extrapolations.

## Sex as a biological variable in treatment-resistant schizophrenia

Sex is a critical determinant of schizophrenia onset, symptom patterns, and treatment response. Men typically experience earlier illness onset and carry a greater burden of negative and cognitive symptoms—patterns that intersect with sex-specific hormonal environments, developmental neurobiology, and stress responsivity (3). Estradiol’s influence on neurogenesis, synaptic pruning, mitochondrial stability, and immune tone is one proposed contributor to later onset and comparatively preserved cognitive trajectories in many women, though these effects remain probabilistic rather than universal (3).

Neuroimaging and translational studies indicate that estrogenic signaling may shape corticolimbic and frontostriatal network integrity through microglial modulation, redox stabilization, and trophic support. These processes, mediated in part by ERβ-linked pathways, have been linked to sex-related differences in vulnerability to treatment resistance (16, 20). Nonetheless, substantial heterogeneity exists within each sex, emphasizing the need for mechanistic, rather than purely demographic, models.

Crucially, estrogenic pathways retain biological relevance in men. ERβ and GPER1 are robustly expressed across male corticolimbic and frontostriatal circuits, and early-illness endocrine changes—including dynamic estradiol–testosterone fluctuations—may influence receptor engagement and downstream intracellular signaling (53). Clinical data further demonstrate that SERMs such as raloxifene can improve cognitive performance and negative symptoms in both men and women, with benefits arising from receptor-level actions rather than feminizing endocrine effects (2). However, sex-stratified analyses reveal variable outcomes, underscoring the need for biomarker-guided interpretation.

Integrating Sex as a Biological Variable (SABV) into TRS research is therefore essential for understanding heterogeneous treatment responses. Sex-linked differences in stress reactivity, HPA axis calibration, and immunoendocrine dynamics influence receptor expression, inflammatory profiles, and mitochondrial signaling (54). Embedding SABV into biomarker development and trial design will support precision-psychiatry approaches that are inclusive of cisgender, transgender, and gender-diverse populations, enabling identification of subgroups most likely to benefit from ERβ- and GPER1-targeted interventions.

## Stem cell and organoid data informing estrogenic modulation in TRS

Human-derived stem cell and organoid systems provide mechanistic insight into how estrogenic signaling influences pathways relevant to schizophrenia and treatment resistance, though most current models are not patient-derived and should be interpreted as informing cellular mechanisms rather than TRS-specific phenotypes.

In forebrain and cortical organoids, estradiol enhances astrocyte–neuron communication, increases dendritic complexity, and supports early network organization—effects that parallel findings from preclinical animal models (6, 55). These cellular features align with domains commonly disrupted in schizophrenia, including altered glial–neuronal signaling and impaired synaptic architecture.

iPSC-derived neuronal cultures clarify receptor-specific pathways. Estradiol promotes neuronal maturation, synaptogenesis, mitochondrial resilience, and calcium-signaling stabilization through ERβ- and GPER1-linked mechanisms (6, 33). These actions occur across both excitatory and inhibitory neuronal subtypes, suggesting that estrogenic modulation engages multiple nodes within dysregulated microcircuits implicated in cognitive and negative symptoms.

Glial models demonstrate complementary receptor-dependent effects. Estradiol modulates astrocytic inflammatory tone—referring to shifts in overall cytokine and signaling profiles—and influences microglial reactivity (16, 20), consistent with evidence that only a subset of individuals with schizophrenia exhibits measurable neuroimmune dysregulation. These findings converge with genetic studies implicating endocrine-linked polymorphisms—including CYP19A1, ESR2, and COMT—in shaping neurodevelopmental trajectories and heterogeneity of symptom expression (46).

Developmental timing is a notable regulatory factor. Early versus late estradiol exposure yields distinct patterns of cortical organization in organoid models (55), reflecting broader evidence that sex steroids sculpt excitatory–inhibitory balance during critical neurodevelopmental windows and may influence vulnerability to neuropsychiatric disorders (56).

While organoids do not recapitulate full *in vivo* circuitry, they provide a scalable platform for mechanistic screening, biomarker discovery, and future evaluation of receptor-selective agents. At present, patient-derived TRS-specific organoid models remain limited, but emerging protocols may enable characterization of TRS-associated cellular phenotypes and preclinical testing of next-generation SERMs or GPER1-biased ligands. Together, these models support—but do not yet confirm—the potential relevance of estrogenic modulation within precision frameworks for TRS.

## The redox–mitochondrial axis in TRS and estrogenic modulation

Mitochondrial dysfunction and oxidative stress are core contributors to TRS, where they impair cognition, weaken synaptic efficiency, and undermine responsiveness to dopamine-targeted therapies. Estrogenic signaling intersects directly with these abnormalities. Through ERβ and GPER1, estradiol enhances mitochondrial biogenesis, stabilizes respiratory-chain function, and engages antioxidant pathways essential for neuronal resilience (23, 30). Rodent SERM studies further support this: selective ERβ and ERα modulators improve sensorimotor gating and synaptic regulation in male mice (57), and GPER1-deficient mice exhibit worsened hippocampal injury and impaired neurogenesis, demonstrating the receptor’s protective role (58). Estradiol also preserves mitochondrial membrane potential and limits reactive oxygen species (ROS) accumulation, thereby supporting synaptic and metabolic integrity (23).

Redox impairments are well documented in schizophrenia, including reduced superoxide dismutase and glutathione peroxidase activity, along with elevated lipid peroxidation—abnormalities associated with mitochondrial DNA damage and bioenergetic inefficiency (27, 59). These molecular disruptions weaken corticolimbic networks and contribute to persistent cognitive and negative symptoms characteristic of TRS.

Estrogenic modulation can buffer these deficits by regulating intracellular calcium homeostasis, reducing mitochondrial permeability-transition pore activation, and optimizing nitric oxide and other redox-sensitive signaling pathways (23, 30). Additional rodent evidence shows that bazedoxifene protects mitochondria from ferroptotic oxidative injury *in vivo* (60) and promotes functional recovery in CNS injury models by suppressing neuroinflammatory and redox-toxic cascades (61). Through receptor-mediated effects that improve ATP production and constrain oxidative load, estradiol promotes neuronal and synaptic resilience across vulnerable circuits.

Glial systems are deeply embedded in this redox–mitochondrial network. Estradiol promotes anti-inflammatory microglial phenotypes, enhances astrocytic antioxidant capacity, and improves neuron–glia metabolic coupling—processes that may reduce chronic inflammatory activation and support dendritic stability (16, 20). Rodent microglial experiments also demonstrate that SERMs reduce inflammatory activation in both mouse and rat glia, supporting a receptor-dependent neuroimmune shift relevant to TRS pathophysiology (57, 58). Disruption of this glial–neuronal antioxidant system contributes to aberrant synaptic pruning, inflammatory persistence, and progressive cognitive decline in TRS.

Hormonal-deficiency states—including menopause and low estradiol bioavailability in males—may diminish redox-buffering capacity, thereby increasing vulnerability to treatment resistance. Meta-analytic evidence suggests that receptor-targeted interventions, including SERMs, can exert therapeutic benefits in part by restoring mitochondrial and antioxidant function (2).

Together, these data position the redox–mitochondrial axis as a hormone-sensitive bottleneck in TRS, where ERβ- and GPER1-selective modulation may stabilize bioenergetics, reduce oxidative injury, and support long-term cognitive outcomes.

## Reframing treatment resistance: hormone receptors as precision targets

TRS affects up to one-third of individuals with schizophrenia and highlights the limitations of dopamine-centric pharmacotherapy. Rather than reflecting isolated dopaminergic insensitivity, TRS encompasses broader disruptions across hormonal, metabolic, and immune domains. Estrogen receptors—particularly ERβ and GPER1—have emerged as potential precision targets within these systems due to their roles in synaptic plasticity, cognitive regulation, mitochondrial stability, and neuroimmune modulation (20, 28, 43).

Clinical and biological evidence converge on this receptor-centered framework. TRS is consistently associated with elevated oxidative stress, mitochondrial inefficiency, and chronic low-grade inflammation—core pathophysiological features of schizophrenia (27, 40). Estrogenic signaling intersects with these domains: ERβ and GPER1 activation can suppress pro-inflammatory cytokine production (20), enhance antioxidant defenses and mitochondrial function (23), and stabilize redox-sensitive trophic cascades linked to synaptic resilience and cognitive performance (25). Raloxifene, a brain-penetrant SERM with documented ERβ/GPER1 engagement, has demonstrated improvements in cognition and negative symptoms in several randomized trials, including among treatment-resistant subsets (2, 8, 28).

Genetic and molecular findings further support the plausibility of receptor-targeted approaches. Variants in ESR2 (encoding ERβ) influence cognitive variability and symptom expression in schizophrenia (62), while CYP19A1 polymorphisms shape sex-linked differences in neuroendocrine regulation relevant to psychosis vulnerability (46). Postmortem analyses also report region-specific alterations in estrogen receptor expression across corticolimbic circuits implicated in schizophrenia (5). Complementary human and non-human primate transcriptomic studies illustrate how hormonal and immune pathways adapt across developmental and environmental contexts, highlighting potential mechanisms through which receptor-level dysregulation may emerge (63).

Reframing TRS as involving disrupted hormone–receptor signaling—rather than solely a failure of dopamine antagonism—positions ERβ- and GPER1-directed modulation as a mechanistically informed adjunctive strategy. These receptor-specific interventions may help restore synaptic integrity, metabolic resilience, and immune balance, offering a biologically grounded path for addressing the multidimensional deficits that characterize the treatment-refractory phenotype.

Historically, estrogenic interventions in psychiatry have centered on women undergoing reproductive transitions, leaving male populations comparatively understudied. However, SERMs provide a mechanism to harness estrogen-linked signaling in a tissue-selective manner without inducing feminizing systemic effects. Across multiple randomized controlled trials, raloxifene has demonstrated improvements in verbal memory, executive functioning, and emotional regulation in individuals with schizophrenia (2, 8, 18). Findings from bipolar disorder offer complementary support: tamoxifen reduces manic symptoms through combined PKC inhibition and estrogen receptor modulation, illustrating the cross-diagnostic relevance of receptor-targeted agents (48, 49). A systematic review further highlights the therapeutic potential of SERMs across mood and psychotic disorders, supporting their applicability for both men and women (64).

Biomarker-guided stratification provides a pathway for identifying individuals most likely to benefit from receptor-targeted approaches. Longitudinal studies show that elevated prenatal cytokine levels predict adult psychosis risk (65), and prenatal maternal stress is associated with heightened cytokine profiles in offspring who later develop psychosis (66). These early immune signatures—reflecting endocrine and inflammatory perturbations during development—may help delineate TRS subgroups characterized by receptor-sensitive neuroinflammatory phenotypes.

Mechanistically, SERMs act as receptor-selective neuromodulators that influence synaptic plasticity, neurotrophic pathways, and mitochondrial efficiency. ERβ activation facilitates coupling between BDNF and 5-HT2A signaling, supporting hippocampal plasticity and affective regulation (67). Raloxifene has been shown to enhance mitochondrial stability, antioxidant capacity, and anti-inflammatory signaling—mechanisms relevant to cognitive restoration and neuroprotection (68). These pathways provide a biologically grounded, non–hormone-replacement route for engaging estrogenic signaling in males, leveraging receptor selectivity rather than systemic endocrine alteration.

Taken together, these findings support a precision-psychiatry framework in which SERM use is tailored to hormonal, immune, and developmental biomarkers. This strategy is sex-informed yet sex-inclusive, aligning receptor biology with individualized clinical profiles. Within this model, SERMs may serve as viable adjunctive interventions for cognition, affect regulation, and neural resilience in treatment-resistant schizophrenia.

## Pharmacokinetics and blood–brain barrier considerations for estrogenic therapies

The clinical utility of estrogenic interventions in schizophrenia depends heavily on achieving adequate central nervous system (CNS) bioavailability while minimizing systemic endocrine effects. Estradiol readily crosses the BBB due to its moderate lipophilicity; however, CNS exposure is constrained by extensive first-pass hepatic metabolism and high-affinity binding to sex hormone–binding globulin (SHBG), which significantly decreases the fraction of free, bioactive estradiol (69), both of which increase systemic load relative to more receptor-selective strategies (70). These pharmacokinetic features contribute to the interest in SERMs as alternatives capable of engaging estrogenic pathways with fewer feminizing effects.

Raloxifene, the most extensively studied SERM in schizophrenia, undergoes rapid phase II glucuronidation and is actively effluxed by P-glycoprotein (P-gp) and multidrug resistance–associated proteins (MRPs), which limits its brain penetration unless transporter activity is genetically reduced or pharmacologically modulated (71). Structural refinements to benzothiophene scaffolds demonstrate that metabolic stability, oxidative metabolism, and redox interactions can be selectively tuned, highlighting the importance of pharmacokinetic optimization for CNS applications (71).

BBB permeability and transporter function are increasingly recognized as dynamic, state-dependent variables rather than fixed barriers. Estradiol has been shown to modulate endothelial tight-junction integrity and enhance BBB resilience under inflammatory or ischemic conditions (72). Parallel work indicates that nanoparticle-mediated drug-delivery systems may bypass efflux pathways and improve CNS bioavailability for estrogenic or SERM-based compounds (73). These findings underscore the need to integrate BBB biology, transporter pharmacodynamics, and hormonal milieu into the design of schizophrenia-specific dosing strategies.

Optimizing CNS delivery while minimizing systemic exposure will likely require individualized approaches. Route of administration (e.g., oral vs. transdermal), metabolic genotype (such as CYP3A4 or UGT1A1 activity), and efflux-transporter expression may all shape achievable brain concentrations and therapeutic windows. Precision-driven pharmacokinetic tailoring may therefore be essential for maximizing the benefits of estrogenic or SERM-based adjunctive therapies while reducing endocrine liabilities in schizophrenia.

## Interaction with antipsychotic pharmacodynamics and side effect profiles

Incorporating estrogenic agents into schizophrenia treatment requires careful consideration of their interactions with dopamine D2 receptor antagonists, the primary pharmacologic approach for reducing positive symptoms. While effective for psychosis, D2 antagonists frequently exacerbate negative symptoms, impair cognition, and contribute to metabolic and motor side effects—including extrapyramidal symptoms (EPS), weight gain, and hyperprolactinemia (74). Because oxidative stress, metabolic imbalance, and neuroinflammation contribute to these burdens, adjunctive agents that buffer redox and inflammatory pathways may help improve overall tolerability and functioning (74).

SERMs may complement these limitations. Adjunctive raloxifene has demonstrated improvements in cognition and negative symptoms even among individuals with suboptimal responses to antipsychotics (75). These effects likely reflect mechanistic complementarity rather than redundancy with D2 blockade: estrogenic signaling influences circuits and cellular domains that dopamine antagonism does not directly engage.

At the circuit level, estrogens modulate dopaminergic dynamics by enhancing mesocortical dopamine tone—which supports executive function—while attenuating subcortical hyperdopaminergia, a contributor to positive symptoms (76). Through ERβ and GPER1, estrogens also regulate D2 receptor expression, influence dopamine transporter (DAT) availability, and activate intracellular cascades that intersect with dopaminergic signaling (76). Early-psychosis research indicates that sex hormone status shapes dopaminergic vulnerability and may contribute to individual variability in antipsychotic response trajectories (77). Functional neuroimaging studies show that raloxifene enhances hippocampal and prefrontal activation during emotional and cognitive tasks, consistent with engagement of non-dopaminergic circuits (77). Preliminary clinical observations also suggest that estrogenic interventions may moderate EPS risk, potentially by stabilizing dopaminergic and inflammatory dynamics (78).

On the immune front, estrogen signaling counteracts pro-inflammatory cytokines such as IL-6 and TNF-α through ERβ and GPER1 and promotes anti-inflammatory glial phenotypes (79). This is clinically relevant because chronic antipsychotic exposure can exacerbate inflammatory signaling and oxidative load—processes in which phosphodiesterase-10A (PDE10A)–related pathways may contribute to both dopaminergic dysregulation and immune activation (80, 81).

The pharmacological characteristics of SERMs further support their adjunctive potential. Their tissue-selective activities differentiate them from systemic estradiol exposure (81), while ERβ-mediated enhancement of BDNF signaling may underlie sustained improvements in cognition, affect regulation, and neural resilience (52, 82).

Collectively, these interactions position SERMs as biologically plausible adjuncts that may:

1. improve cognitive and affective symptoms,
2. buffer metabolic, inflammatory, and oxidative stress associated with antipsychotic therapy, and
3. reduce certain side-effect burdens by stabilizing dopaminergic and neuroimmune systems.

These mechanisms support the integration of estrogenic modulation into precision frameworks for treatment-resistant schizophrenia.

## Developmental timing and critical period sensitivity

Schizophrenia follows a neurodevelopmental trajectory shaped by sensitive periods in which hormones, synaptic pruning, and inflammatory cues influence long-term circuit architecture. Estradiol exerts stage-specific effects on neural plasticity, making the timing of intervention an important determinant of therapeutic potential. Preclinical studies show that perturbing molecular pathways involved in adolescent maturation—particularly those affecting dopaminergic and corticostriatal signaling—can lead to enduring deficits in cognition, motivation, and executive functioning that resemble treatment-resistant phenotypes (50).

Adolescence is marked by extensive dendritic remodeling, refinement of corticolimbic circuits, and regulation of intracellular second-messenger cascades that support learning and emotional regulation. Disruption of pathways interfacing with estrogen receptors—including phosphodiesterase-dependent modulation of cAMP/PKA and MAPK/ERK signaling—impairs executive function and stress responsivity in preclinical models (50, 83). Because ERβ and GPER1 interact with these intracellular systems, endocrine perturbations during adolescence may have lasting consequences for circuit maturation and later vulnerability.

Early adulthood, the developmental window in which schizophrenia symptoms typically consolidate, represents another phase of heightened plasticity. During this period, interventions engaging estrogen receptor pathways may help stabilize synaptic organization, support mitochondrial function, and buffer against the progression of cognitive and negative symptoms. Receptor-targeted pharmacology suggests that compounds with selective action on estrogen-responsive pathways—including SERMs and selective estrogen receptor degraders (SERDs)—can modulate intracellular cascades relevant to resilience and long-term circuit stabilization (84).

Related evidence links estradiol-sensitive trophic cascades, such as BDNF–CREB signaling, to the developmental refinement of prefrontal and hippocampal networks (85). Because BDNF activity is closely associated with cognitive and affective outcomes in schizophrenia, developmental disruptions in these pathways may contribute to later treatment resistance.

Collectively, these findings support a developmental framework in which hormone-sensitive circuits show age-dependent vulnerability—and thus age-dependent therapeutic receptivity. Precision-timed estrogenic or SERM-based interventions may have the greatest impact when aligned with these neurodevelopmental windows, offering a biologically informed strategy to influence trajectories in individuals at elevated risk for treatment-resistant schizophrenia.

## Genetic and hormonal stratification: COMT, SHBG, and hormone profiling

COMT remains one of the most clinically informative genetic markers for understanding dopamine variability in schizophrenia. Functional COMT variants—including those that influence cortical dopamine turnover—shape cognitive performance and interact with neurobiological risk pathways relevant to psychosis vulnerability (86). These genotype-linked effects may also be modulated by hormonal context: endocrine states influence biomarker expression, stress responsivity, and circuit-level vulnerability across psychiatric conditions, suggesting that COMT effects are partly contingent upon broader hormonal and immune environments (87).

Endocrine markers provide a second axis for biological stratification. Circulating estradiol, luteinizing hormone (LH), and sex hormone–binding globulin (SHBG) jointly determine the proportion of bioavailable estradiol, a variable that may inform responsiveness to receptor-targeted interventions. Evidence from translational neuroimmunology indicates that ERβ-responsive ligands exert differential effects depending on baseline hormonal milieu, aligning with clinical findings that hormone-sensitive pathways modulate overall inflammatory tone—reflected in cytokine and glial-signaling profiles—alongside mitochondrial function and synaptic resilience (88). These results support incorporating peripheral hormone profiles into augmentation strategies for schizophrenia.

Receptor-level biological variability adds further specificity. Preclinical work demonstrates that sex-linked differences in ERβ signaling—and its interaction with BDNF-related trophic pathways—shape behavioral and cognitive outcomes relevant to psychosis (89). Clinical observations similarly indicate that cognitive responses to raloxifene are not uniform across individuals and may depend on receptor expression, downstream signaling efficiency, or broader endocrine context (47). These findings highlight the importance of stratifying estrogenic interventions based on receptor biology rather than assuming uniform benefit.

Genotype–hormone interactions may be particularly relevant during aging or endocrine transition. Work in both healthy and schizophrenia populations shows that the cognitive impact of COMT variants shifts across hormonal states, including during menopause, when estradiol levels decline and dopaminergic vulnerability increases (62). These findings suggest that TRS may emerge partly from mismatches between receptor biology, endocrine tone, and pharmacologic mechanism.

Within this framework, genetic polymorphisms (e.g., COMT variants), endocrine markers (e.g., SHBG-modulated estradiol availability), and receptor-linked signatures together support a precision-medicine approach. Such multimodal stratification can guide the use of estrogenic agents—including raloxifene—as targeted adjuncts for cognitive and negative symptoms across sexes, aligning therapeutic mechanisms with individualized neurobiological profiles.

Algorithm 1 — Biomarker-Guided Patient Selection for Estrogenic Adjuncts in TRS.

Step 1 — Baseline Clinical Phenotype.

Identify patients who meet.

- Diagnostic criteria for TRS (nonresponse to ≥2 adequate antipsychotic trials)
- Persistent negative or cognitive symptoms
- Limited benefit from dopamine-targeted interventions

Step 2 — Endocrine Profile (Peripheral Biomarkers).

Obtain baseline hormone panel.

• Estradiol, testosterone, LH/FSH, and SHBG
 – Low bioavailable estradiol or elevated SHBG → favors SERM over estradiol replacement
• Assess metabolic status (lipid panel, HbA1c) prior to initiating estrogenic adjuncts

Step 3 — Immune and Redox Markers.

Evaluate inflammatory and oxidative stress indices.

• Cytokines: IL-6, TNF-α, IL-10
• Redox markers: glutathione (GSH), oxidized lipids, 8-OHdG
 – Elevated inflammatory/redox burden predicts greater potential responsiveness to ERβ/GPER1-mediated modulation

Step 4 — Genetic Stratification.

Prioritize gene variants linked to receptor or dopamine–estrogen interactions.

• COMT Val158Met
 – Met carriers show greater estrogen-sensitive dopaminergic modulation
• ESR2 (ERβ) and CYP19A1 (aromatase) variants
 – May influence receptor sensitivity, neurosteroidogenesis, and treatment response

Step 5 — Receptor Distribution/Neurocircuit Indicators *(optional; research-stage)*.

In specialized settings, consider advanced biomarkers:

- PET or CSF indices of ERβ/GPER1 expression
- fMRI markers of hippocampal–prefrontal underactivation
- Organoid/iPSC phenotyping (glial tone, calcium dynamics, mitochondrial stability)

Step 6 — Integrative Decision Node.

A patient is a candidate for adjunctive SERM therapy when.

✓ TRS with predominant negative or cognitive symptoms
✓ Evidence of inflammatory or oxidative stress burden
✓ Low bioavailable estradiol or elevated SHBG
✓ Genetic or endocrine profile consistent with ERβ/GPER1 responsiveness
✓ No contraindications (e.g., high thromboembolic risk requiring exclusion)

Step 7 — Monitoring Strategy.

Establish longitudinal tracking.

- Baseline and follow-up metabolic panel, endocrine markers, and coagulation risk
- Symptom monitoring using PANSS-N, MATRICS, and standardized cognitive assessments
- Adjust SERM dosing based on clinical progression and biomarker shifts

## Ethical and gender-inclusive considerations in hormonal psychiatry

As estrogenic strategies are integrated into schizophrenia care—particularly for TRS—ethical and gender-inclusive considerations become essential. Early hormonal interventions in psychiatry focused almost exclusively on cisgender women undergoing reproductive transitions, yet both male and female brains express ERβ and GPER1, and these receptors modulate circuits implicated in psychosis, cognition, and affective regulation (75, 76). Ensuring equitable application of receptor-targeted strategies requires attention to structural biases and to the lived experiences of transgender and gender-diverse individuals, who remain significantly underrepresented in psychiatric research (77).

A key ethical distinction lies in differentiating systemic feminization from CNS-selective receptor modulation. SERMs such as raloxifene activate ERβ and GPER1 in the brain while exerting antagonistic or neutral effects in peripheral estrogen-responsive tissues. This receptor selectivity enables augmentation of antipsychotic therapy without producing feminizing endocrine effects (28). For cisgender men and transmasculine individuals, transparent communication about this pharmacologic specificity is central to fostering informed and identity-affirming consent.

Gender-affirming hormone therapy (GAHT) introduces additional layers of complexity. Testosterone modulates receptor distribution, CYP19A1 activity, and intracellular signaling pathways relevant to dopamine and glutamate regulation (77), whereas estradiol-based GAHT alters CYP450 metabolism, SHBG levels, and neurosteroid balance (78). These changes may influence SERM pharmacokinetics or CNS efficacy, highlighting the need for coordinated care between psychiatry, endocrinology, and primary care. Integrated monitoring ensures that estrogenic adjuncts support psychiatric stability without disrupting GAHT regimens or identity-related therapeutic goals.

Research practices must also evolve toward inclusivity. Many psychiatric studies continue to rely on binary sex classifications, limiting mechanistic insight into endocrine–neural interactions. Strategies such as gender-inclusive eligibility criteria (79), disaggregated outcomes by gender identity (80), and intentional recruitment of transgender participants are necessary to build an evidence base that reflects real-world populations. These steps align with broader precision-psychiatry goals by reducing sampling bias and clarifying how receptor biology varies across hormonal contexts.

Finally, equitable access must be considered. As receptor profiling, endocrine panels, and biomarker-guided stratification become more central to TRS care, it is important that these tools do not reinforce existing disparities or binary assumptions. Embedding gender-expansive principles within precision psychiatry ensures that estrogenic interventions remain mechanistically grounded while affirming diverse identities, supporting ethical and scientifically rigorous implementation across populations.

## Comparative efficacy and safety of different SERMs

SERMs vary widely in receptor affinity, metabolic stability, and CNS penetration—features that determine their clinical utility in schizophrenia. Among currently available agents, raloxifene, tamoxifen, and bazedoxifene represent the most informative comparators for psychiatric application.

Raloxifene holds the strongest evidence base in schizophrenia. Multiple randomized controlled trials demonstrate improvements in verbal memory, executive function, and negative symptoms across postmenopausal women and mixed-sex cohorts (2, 8, 18). Neuroimaging studies further show increased hippocampal and inferior frontal activation during emotional and cognitive tasks under raloxifene treatment (19, 21), consistent with CNS engagement of ERβ- and GPER1-linked pathways. Its tissue-selective pharmacology minimizes feminizing endocrine effects; however, raloxifene is a P-gp substrate, which limits BBB penetration. GPER1 activation engages PI3K/Akt, ERK/MAPK, and calcium–calmodulin pathways, supporting synaptic and metabolic adaptability (84). These transport constraints may be partially offset during neuroinflammatory states or addressed through optimized delivery platforms.

Tamoxifen, though primarily used in oncology, also exhibits CNS-relevant SERM properties. Preclinical studies show that tamoxifen modulates dopaminergic activity, improves sensorimotor gating, and attenuates hyperlocomotor phenotypes in rodent models relevant to psychosis (48). Clinically, tamoxifen’s antimanic efficacy in bipolar disorder is attributed to dual mechanisms: PKC inhibition and estrogen receptor modulation (48, 49). These cross-diagnostic effects underscore the relevance of estrogenic and intracellular signaling pathways across severe mood and psychotic disorders. However, tamoxifen’s long-term psychiatric use is limited by safety concerns—including thromboembolic risk and endometrial stimulation—making it less suitable for chronic schizophrenia management.

Bazedoxifene, a third-generation SERM, has not yet been directly evaluated in schizophrenia but possesses several mechanistic advantages. It demonstrates ERβ-selective engagement, attenuates inflammatory signaling, and exhibits a more favorable metabolic and vascular safety profile compared with earlier SERMs (85). Bazedoxifene also shows reduced endometrial stimulation, an important consideration for patients with elevated cardiometabolic or hormonal baseline risk. Emerging preclinical evidence suggests that bazedoxifene is capable of BBB penetration and may modulate neuroimmune pathways relevant to schizophrenia pathophysiology (85), making it a strong candidate for translational investigation.

Taken together, these comparisons suggest that raloxifene currently offers the most robust clinical support; tamoxifen provides mechanistic validation and cross-diagnostic insight; and bazedoxifene represents a promising next-generation agent with improved safety and potentially superior receptor selectivity. Considering SERMs as a pharmacological class—rather than focusing exclusively on raloxifene—broadens the therapeutic landscape for receptor-selective estrogenic strategies in TRS (85). To clarify how different SERMs may align with receptor-specific and safety considerations in schizophrenia, **Table 3** summarizes comparative pharmacologic profiles—including receptor selectivity, CNS penetrance, clinical advantages, and key limitations—across the three most relevant agents.

**Table 3:** Comparative profiles of SERMs relevant to schizophrenia.

SERM	Receptor selectivity	CNS penetrance	Key advantages	Limitations	Supporting evidence
Raloxifene	Partial ERβ agonist; ERα antagonist	Moderate; P-gp substrate	Cognitive improvement, negative symptom reduction, minimal feminization	Efflux-limited BBB penetration; mixed effects across trials	(2, 8, 18, 75, 81)
Tamoxifen	Mixed ER modulator; PKC inhibitor	High CNS penetration	Antimanic effects; dopaminergic modulation; cross-diagnostic relevance	Thromboembolic risk; endometrial effects; long-term safety concerns	(48–50, 82)
Bazedoxifene	ERβ-biased, third-generation	Predicted moderate CNS entry	Favorable metabolic & vascular profile; minimal thrombotic risk	Psychiatric trials lacking; underexplored	(83, 85, 86)

**Table 3**. Comparative pharmacologic characteristics of SERMs relevant to schizophrenia. Receptor selectivity reflects predominant engagement of ERβ, ERα, or mixed mechanisms. CNS penetrance denotes relative BBB permeability based on clinical and preclinical data. Key advantages and limitations highlight domains with therapeutic promise or safety considerations. References correspond to studies evaluating cognitive, affective, metabolic, or mechanistic outcomes in psychiatric and translational models.

## Safety and monitoring considerations for estrogenic adjuncts in schizophrenia

Integrating estrogenic agents into schizophrenia treatment requires careful attention to safety, pharmacokinetic interactions, and individualized risk stratification. Although SERMs and estradiol engage mechanistically complementary pathways to dopamine antagonists, both classes influence vascular, metabolic, and endocrine systems in ways that necessitate structured monitoring.

### Thromboembolic and vascular risks remain central considerations

Tamoxifen and older SERMs carry elevated risk for venous thromboembolism (VTE) and endometrial stimulation, particularly in patients with pre-existing cardiovascular or coagulation vulnerabilities (50, 85). Raloxifene exhibits a more favorable vascular and endometrial profile (81), but caution is still warranted in individuals with smoking history, obesity, prolonged immobility, or genetic thrombophilia. VTE screening therefore represents a foundational component of patient selection.

### Drug–drug and metabolic interactions require coordinated management

SERMs interact with CYP450 isoenzymes—most notably CYP3A4 and CYP2D6—which may influence metabolism of commonly prescribed antipsychotics (78). Estradiol additionally modulates transporter activity (e.g., P-gp), undergoes high-affinity binding to SHBG, and exhibits nonlinear hepatic metabolism (70). These factors can alter systemic exposure to both estrogenic agents and antipsychotics, reinforcing the need for therapeutic monitoring and dose adjustments when co-administered in polypharmacy contexts.

### Endocrine effects should be interpreted through a sex-informed yet gender-inclusive lens

Because SERMs selectively engage ERβ and GPER1 without increasing circulating estradiol, they do *not* induce feminizing endocrine effects in men (28, 75). However, testosterone therapy in transmasculine individuals may alter receptor distribution and CYP450 activity (77), while estradiol-based regimens in transfeminine patients may modulate neurosteroid availability and SERM metabolism (78). Collaborative management between psychiatry and endocrinology is essential to ensure safety and affirming care across gender identities.

### Neuroimmune and metabolic factors interact with antipsychotic side effect profiles

Chronic antipsychotic treatment increases oxidative stress and metabolic burden, domains in which estrogenic pathways exert anti-inflammatory and antioxidant effects (74, 79). Conversely, antipsychotic-induced cytokine changes may alter BBB function and influence SERM penetrance (73), suggesting that dosing strategies may need to adapt to inflammatory state or illness stage.

Practical monitoring recommendations include:

- Baseline and interval lipid panel, liver enzymes, and coagulation history
- Screening for VTE risk factors (smoking, immobility, obesity, prior thrombotic events)
- Assessment of CYP450-mediated interaction potential in polypharmacy
- Hormonal profiling (estradiol, testosterone, SHBG) when employing biomarker-based stratification
- Monitoring for rare mood effects reported with tamoxifen (48, 49)

Within these parameters, raloxifene remains the safest and most feasible estrogenic adjunct for schizophrenia, with the most favorable balance of CNS efficacy and systemic tolerability. Careful patient selection, transparent counseling, and structured monitoring are essential to ensure that receptor-selective modulation enhances cognition and affect while maintaining systemic safety.

## Future directions: brain-selective estrogen mimetics and designer SERMs

The next generation of estrogenic psychiatry will rely on compounds engineered for CNS selectivity—agents capable of reproducing estradiol’s cognitive, neuroprotective, and anti-inflammatory benefits while minimizing systemic endocrine effects. This shift reflects emerging biomarker-driven frameworks emphasizing receptor specificity, endocrine context, and individualized risk profiles (88). Translational studies using ERβ-selective ligands demonstrate robust neuroprotective actions in neuroinflammatory models, supporting the feasibility of receptor-targeted approaches optimized for CNS signaling (89).

Progress in SERM development is moving beyond raloxifene toward compounds with more refined pharmacodynamic properties. Although tamoxifen has provided key insights into PKC-linked neuromodulatory mechanisms, its systemic liabilities restrict long-term use in psychiatric populations. Preclinical findings linking estrogen–BDNF interactions and receptor-mediated plasticity to cognitive and affective improvements (47, 90) bolster the rationale for developing safer analogues with enhanced receptor selectivity and reduced endocrine risk. Bazedoxifene—characterized by an improved vascular and metabolic profile and lower endometrial stimulation—represents an underexplored candidate whose tolerability may be advantageous for chronic adjunctive therapy in schizophrenia (62).

One of the most promising frontiers is the design of receptor- and pathway-biased ligands. GPER1-selective or ERβ-biased compounds could selectively activate rapid intracellular cascades such as PI3K/Akt and MAPK/ERK—pathways central to synaptic remodeling, mitochondrial stabilization, and glial regulation—while avoiding transcriptional activation in reproductive tissues (62, 90). These ligands offer the potential for brain-specific estrogenic benefits with substantially reduced feminizing or proliferative side effects.

A complementary strategy targets intracellular signaling systems that converge with estrogenic pathways. PDE10A, a striatal enzyme integrating dopaminergic and glutamatergic information through cAMP/cGMP modulation, has strong translational relevance for negative and cognitive symptoms in schizophrenia (50, 83). Although direct estrogen–PDE10A interactions remain uncharacterized, both systems converge on shared second-messenger architectures, raising the possibility of synergistic or combined pharmacologic approaches in future drug development (91).

Finally, tissue-selective estrogen complexes (TSECs)—originally developed for menopausal therapy—offer a hybrid platform capable of combining CNS-selective estrogenic benefits with peripheral receptor antagonism. By pairing estrogens with SERMs, TSECs can engage ERβ-mediated neuroprotective pathways while concurrently blocking proliferative estrogen receptor activity in peripheral tissues (62, 92). Adapting TSECs for schizophrenia could yield dual-mechanism compounds optimized for cognitive enhancement, anti-inflammatory action, CNS targeting, and long-term safety.

## Technological innovation in estrogenic drug delivery and scaffold design

Nanoparticle-mediated drug delivery is rapidly expanding the feasibility of brain-targeted estrogenic interventions. Cyclodextrin-based nanosystems enhance solubility and molecular stability, while lipid-derived nanoparticles provide flexible encapsulation platforms for SERMs such as raloxifene and bazedoxifene (73). PEGylated nanoparticles further improve pharmacokinetic endurance and cross the BBB via ligand-mediated transcytosis, protecting compounds from enzymatic degradation and increasing CNS penetration (73). These delivery strategies are especially relevant for SERMs subject to extensive hepatic metabolism or active efflux via P-glycoprotein, offering a route to enhance central bioavailability while reducing systemic hormonal exposure.

Parallel advances in synthetic scaffold engineering are generating non-steroidal molecules capable of reproducing estrogenic activity with high selectivity for ERβ and GPER1. Rationally designed benzothiophene scaffolds, for example, permit preferential engagement of neuroprotective signaling pathways while minimizing peripheral receptor activation (71). By uncoupling CNS benefits from systemic endocrine effects, these scaffolds lower risks of feminization, thromboembolism, or proliferative stimulation, thereby broadening their suitability for psychiatric indications. Development of ERβ-selective neuroprotective ligands in other CNS disorders further highlights their translational promise (87).

Emerging translational tools now connect ligand design directly to patient-specific biology. Experimental PET ligands targeting estrogen receptors have been developed in preclinical models, providing proof-of-concept for receptor-specific molecular imaging, while iPSC- and organoid-derived platforms enable individualized testing of ERβ- and GPER1-selective compounds (6, 55). Although validated human PET tracers for ERβ or GPER1 are not yet available, ongoing ligand-development efforts highlight the feasibility of future *in vivo* receptor mapping. These approaches create a mechanistic bridge between molecular pharmacology, receptor-expression dynamics, and circuit-level dysfunction.

Together, advances in nanotechnology, scaffold design, and biomarker-driven translational tools mark a shift toward precision estrogenic therapeutics—agents optimized for CNS selectivity, mechanistic specificity, and long-term safety in treatment-resistant schizophrenia.

## Conclusion

TRS remains a major clinical challenge, particularly in populations where cognitive deficits (47) and negative symptoms (26) are prominent. Estrogenic strategies—especially SERMs that engage ERβ and GPER1—offer mechanistically grounded avenues to target these domains. Evidence from preclinical models (88) and translational human studies (18) demonstrates that estrogenic signaling can stabilize corticolimbic circuitry, modulate neuroimmune tone, and enhance mitochondrial and synaptic resilience, all of which are implicated in treatment resistance.

Clinical results, however, remain heterogeneous. Meta-analytic work shows variable improvements in cognition and negative symptoms, with some randomized trials reporting meaningful gains and others yielding modest or nonsignificant effects (44, 47). These discrepancies likely reflect symptom heterogeneity, endocrine context, receptor-genetic variation, and differences in SERM pharmacodynamics, highlighting the need for cautious interpretation and signaling that current evidence is preliminary but promising.

Among existing agents, raloxifene is the best-supported candidate. Controlled trials report improvements in cognitive performance (8) and affective or neuroimmune domains (46), while its tissue-selective pharmacology minimizes feminizing endocrine effects. Complementary mechanistic data—including immune regulation (40), neurotransmitter modulation (22), and stress-buffering effects within corticostriatal circuits—mechanisms supported in both neurodegenerative and psychiatric models (88)—support its potential role as an adjunctive intervention for TRS.

A precision framework will likely determine the success of estrogenic approaches. Biomarkers including ESR2 and COMT variation (46, 47), CYP19A1 polymorphisms (46), and circulating measures such as SHBG or inflammatory cytokines (42) offer tools to identify patients whose receptor biology or endocrine milieu favors responsiveness. Developmental timing may further shape therapeutic windows: adolescence—marked by intense cortical pruning (12)—and early adulthood, when symptoms consolidate (15, 88), represent potential periods of heightened sensitivity to receptor-based modulation.

Future work must also prioritize gender-inclusive and ethically grounded research practices. Trials historically limited to cisgender women overlook the widespread expression of ERβ and GPER1 across sexes. Inclusion of transgender (77–79) and non-binary participants (79) is essential to ensure that receptor-informed psychiatry is both scientifically rigorous and socially equitable.

Taken together, estrogenic and SERM-based adjuncts represent a compelling, mechanistically precise, and developmentally informed strategy for TRS. While further research is needed to define optimal patient selection and timing, the emerging evidence base points toward a more biologically grounded and individualized future for schizophrenia treatment.

## Data Availability

The original contributions presented in the study are included in the article/supplementary material. Further inquiries can be directed to the corresponding author.
